# Supplementary material for: Little skate genome provides insights into genetic programs essential for limb-based locomotion
Source: eLife. 2022 Oct 26;11:e78345. doi: 10.7554/eLife.78345 (PMC9605692; doi:10.7554/eLife.78345)
Supplement: Supplementary file 8. — T7 promoter sequence is indicated in blue [file elife-78345-supp8.docx]

| **Additional Data 9. Oligonucleotide sequences used in generating *in situ* probes**  T7 promoter sequence is indicated in blue | | |
| --- | --- | --- |
| Gene | Oligonucleotide sequence | |
| Mouse | 5’ -> 3’ | |
| *Eya1* | Fwd | ggcagacacacatctatttttc |
|  | Rev | TAATACGACTCACTATAGGGATTCCAAGGCATGATGCAGTG |
| *Uchl1* | Fwd | agccgatggagattaaccccg |
|  | Rev | TAATACGACTCACTATAGGGGCTGCTTTGCAGAGAGCCAC |
| *Slc5a7* | Fwd | ccatcctgtcggcgagttc |
|  | Rev | TAATACGACTCACTATAGGGCCGGACTGGAATCAACATCAAG |
| *Coch* | Fwd | cacatgcccaactggtttggc |
|  | Rev | TAATACGACTCACTATAGGGTTGCTGGGATTCTAAGAAGTCTC |
| *Dnmbp* | Fwd | gggtgccatcgacagaacag |
|  | Rev | TAATACGACTCACTATAGGGCCGGATATAGTTGGATGGGAC |
| *Ctxn1* | Fwd | agttcggcgtggacactatc |
|  | Rev | TAATACGACTCACTATAGGGCACCAACGCATAGTCGAACTG |
| *Spint1* | Fwd | GGGCTCCTTCCCACGCTGGTAC |
|  | Rev | TAATACGACTCACTATAGGGTCCTCTGTGGTGGAAACAGTG |
| *Clmp* | Fwd | AGGACGCTATGTCTGGAGCC |
|  | Rev | TAATACGACTCACTATAGGGGACAGTTTGGAAGGCTTTGCTC |
| *Ppp1r1c* | Fwd | GAGCCCAATAGCCCCAAAAAG |
|  | Rev | TAATACGACTCACTATAGGGGGTTCCACTTCTCTTCTCTTTC |
| *Serinc1* | Fwd | CGTGCTATTCTTTGTCTACTAC |
|  | Rev | TAATACGACTCACTATAGGGCCCGATTTGTAAGAACAAGTGG |
| *Etv5* | Fwd | ggatgcctggtcctccagcac |
|  | Rev | TAATACGACTCACTATAGGGAGGCTGCTGCAGCGATCCATG |
| *Alcam* | Fwd | ggaggtgaccaagcagattggcg |
|  | Rev | TAATACGACTCACTATAGGGCATATTACCAAGGTCCTTGTTTAC |
| *Thra* | Fwd | GGCGAAAATTCCTGCCGGATGAC |
|  | Rev | TAATACGACTCACTATAGGGGATCCTCAAAGACCTCCAGGAAG |
| *Lypd1* | Fwd | TGTCAGAAAGAAGTGATGGAGC |
|  | Rev | TAATACGACTCACTATAGGGGCAGTGTGCCAAGCAGAGGGC |
| *Cib2* | Fwd | GGGAACAAGCAGACCATCTTCAC |
|  | Rev | TAATACGACTCACTATAGGGGATTCGAATGTGGAAGGTGCTG |
| *Fpgs* | Fwd | TGTGCAGGCCTGTGTGCACTG |
|  | Rev | TAATACGACTCACTATAGGGGAGGGATCCAGCAGTTTCAG |
| *Tent5c* | Fwd | AACAAGAACGGGAGGAACGTG |
|  | Rev | TAATACGACTCACTATAGGGGGTAATTGGAGGGTGCGCAATG |
| *Qsox1* | Fwd | CTCTTCTGCATCTAGACACAC |
|  | Rev | TAATACGACTCACTATAGGGTTCTCAGCCTGGCCCGAAAG |
| *Wsb1* | Fwd | GGAAGCTGGAAGGTCATCACC |
|  | Rev | TAATACGACTCACTATAGGGACCGCGGTAGGAGAGAAACGC |
| *Rab18* | Fwd | GACGAGGACGTGCTGACCAC |
|  | Rev | TAATACGACTCACTATAGGGTAGCACAGAGCAGTAACCGC |
| *Atp6v1d* | Fwd | CGGGCAAAGACCGGATTG |
|  | Rev | TAATACGACTCACTATAGGGTCGAACAGCAGGTCTTCATCC |
| *Skp1* | Fwd | CCTACGATAAAGTTGCAGAGTTC |
|  | Rev | TAATACGACTCACTATAGGGCTTCTCTTCACACCATTGGTTC |
| *Psma6* | Fwd | CCGACTCTACCAAGTAGAATATGC |
|  | Rev | TAATACGACTCACTATAGGGACAAGGTGAGCGTCAATCTCTG |
| *Spcs1* | Fwd | TCGGAGACATCCGCTTCC |
|  | Rev | TAATACGACTCACTATAGGGCTGTCCCCTGATTTCTTGTCTTC |
| *Mad2l1* | Fwd | GCACAGCAGCTCGCCCGAGAG |
|  | Rev | TAATACGACTCACTATAGGGGACAGGGGTTTTGTAGGCCAC |
| *Slc2a8* | Fwd | CCTCATCATCGGCATTTCCC |
|  | Rev | TAATACGACTCACTATAGGGCGTCCCTCGAAATGGGCTGTG |
| *Klhl36* | Fwd | TAAACAGTGGATCAAGGTGG |
|  | Rev | TAATACGACTCACTATAGGGGACACACCCGCAATGGCG |
| Gene | Oligonucleotide sequence | |
| Skate | 5’-3’ | |
| *Eya1* | Fwd | gccacagatggctttcATGC |
|  | Rev | TAATACGACTCACTATAGGGCAAGTACTCCAGTTCCAAGG |
| *Uchl1* | Fwd | TGGCTTCGAGCTGGAAGTTC |
|  | Rev | TAATACGACTCACTATAGGGATTTGCACAGAGCCACTGCAG |
| *Slc5a7* | Fwd | CTTGCTTTCAGACAAGAGGC |
|  | Rev | TAATACGACTCACTATAGGGTGCTTCCTTATTGGTGAAAGC |
| *Coch* | Fwd | GATGGCCCTCGGATGACGTGG |
|  | Rev | TAATACGACTCACTATAGGGTATCAGGCACCAACTTGTGAAC |
| *Dnmbp* | Fwd | CTCGGACATCTCGGTGGGAAG |
|  | Rev | TAATACGACTCACTATAGGGGGCACGTAGCCCTGCTTGCCG |
| *Ctxn1* | Fwd | GACGATGCCGCCGCCATGAATG |
|  | Rev | TAATACGACTCACTATAGGGCACCAAGGCGTAGTCAAACTGzzzz |
| *Spint1* | Fwd | CCATAACCGTCCTCTCGCCAG |
|  | Rev | TAATACGACTCACTATAGGGGACGGGCTTCGAGTCCAGGTG |
| *Clmp* | Fwd | TGCAGGCGACAGATTCTGGAC |
|  | Rev | TAATACGACTCACTATAGGGCACAGTCTGGAAAGCCCTGG |
| *Ppp1r1c* | Fwd | GAAGATTCAATTTGCTGTGCCG |
|  | Rev | TAATACGACTCACTATAGGGGTTGGTCTTTGGTAACCACTC |
| *Serinc1* | Fwd | ATGGAAGAGGGCAATTCTAGG |
|  | Rev | TAATACGACTCACTATAGGGCACGAGTTGTCAAAACAAGTGG |
| *Etv5* | Fwd | CATACCGGCGCCAGTCGGGAG |
|  | Rev | TAATACGACTCACTATAGGGCACTGATCCGTGTCCAGCAGG |
| *Alcam* | Fwd | GGAGATGAGAAAGCTGTGGG |
|  | Rev | TAATACGACTCACTATAGGGCACAGCTGAGGAGCCTGATG |
| *Thra* | Fwd | ACCAACGCACAAGGCAGCCAC |
|  | Rev | TAATACGACTCACTATAGGGCCTGATCTTCAAATACCTCC |
| *Lypd1* | Fwd | CAGATCTTCATTTACACAACTTTC |
|  | Rev | TAATACGACTCACTATAGGGGTTAAGAGTGTTGAAGTGAGCATTC |
| *Cib2* | Fwd | GCTCCTCACATAGTGCCAATGG |
|  | Rev | TAATACGACTCACTATAGGGGATCCGAATGTGAAATGTGCTG |
| *Fpgs* | Fwd | CATATTACATTGATGGTGCTC |
|  | Rev | TAATACGACTCACTATAGGGGCCCACTAGGTGGAGGCTG |
| *Tent5c* | Fwd | CTCCCTCCTGTTTTACTACG |
|  | Rev | TAATACGACTCACTATAGGGGGGAGCCAGGTAGGGTACGAC |
| *Qsox1* | Fwd | AGTGAGGATCCAAAGTTCCC |
|  | Rev | TAATACGACTCACTATAGGGCCACTTCCTTTCCTTGCTATG |
| *Wsb1* | Fwd | ACTGGCTTAAGCAATGGACGC |
|  | Rev | TAATACGACTCACTATAGGGGCCTTCACATTAAGCAGTTCAC |
| *Rab18* | Fwd | AGGGAGCGATGGACGAAGAC |
|  | Rev | TAATACGACTCACTATAGGGACTGAGCAATATCCACCAC |
| *Atp6v1d* | Fwd | CCGCTGGTGATAAAACTGAAG |
|  | Rev | TAATACGACTCACTATAGGGGATTGCATGAATCCACTCAGATC |
| *Skp1* | Fwd | CCTTCCATTAAGCTCCAGAGC |
|  | Rev | TAATACGACTCACTATAGGGCACACCACTGATTCTCCTTGC |
| *Psma6* | Fwd | TCGCGGGGATCGAGCGCCGGCTTC |
|  | Rev | TAATACGACTCACTATAGGGATCTCTCTCTGCCAGAGCAACC |
| *Spcs1* | Fwd | GCTGGCGAGAGCGGCGGAGAG |
|  | Rev | TAATACGACTCACTATAGGGATGCTTTTTAGGTTTCTTGGCAC |
| *Mad2l1* | Fwd | GCATCAACAGTATTCTGTACCAA |
|  | Rev | TAATACGACTCACTATAGGGGTACGCAACCAAACTGTTTAC |
| *Slc2a8* | Fwd | GCCTTTCCTTATTGGGATATTTC |
|  | Rev | TAATACGACTCACTATAGGGGGTCCTCTAAAGAGGGCCTC |
| *Klhl36* | Fwd | TAATCAGTGGCTCAAGGTTGC |
|  | Rev | TAATACGACTCACTATAGGGCCACAGTCTTGGCGTATAGTC |
| Gene | Oligonucleotide sequence | |
| Chick | 5’-3’ | |
| *Eya1* | Fwd | agatgattttcaatttggcagac |
|  | Rev | TAATACGACTCACTATAGGGGAAGGGCCATAAGGTCAGAG |
| *Uchl1* | Fwd | gcgtggcagcccatggagatc |
|  | Rev | TAATACGACTCACTATAGGGGGCAGACTTGCAGAAAGCCAC |
| *Slc5a7* | Fwd | catgtcatcagctgactcttc |
|  | Rev | TAATACGACTCACTATAGGGGGCTTCCTTGTTTGTGAAAGTTG |
| *Coch* | Fwd | tgtgttcattgtatctgtagc |
|  | Rev | TAATACGACTCACTATAGGGCAGGAACCATCTGCTCCAATC |
| *Dnmbp* | Fwd | agctcctttctgaagccctac |
|  | Rev | TAATACGACTCACTATAGGGGTACTCTGTCTTCCGGATG |
| *Ctxn1* | Fwd | gatgcatcgacgatggattatg |
|  | Rev | TAATACGACTCACTATAGGGCACCAAGGCGTAGTCAAACTGG |
| *Spint1* | Fwd | ATGACCCAACTCTTCAGCAGTG |
|  | Rev | TAATACGACTCACTATAGGGGACTGGTTTGGTGGCACCGTTG |
| *Clmp* | Fwd | GCTGGGCAGTACGAATGGGCTC |
|  | Rev | TAATACGACTCACTATAGGGCACGGTCTGGAACGCTCGGC |
| *Ppp1r1c* | Fwd | TGGAATAAGGGCGCAGCTCAGC |
|  | Rev | TAATACGACTCACTATAGGGTCCCTGGACAGTACCTTTGAGGG |
| *Serinc1* | Fwd | CGTTTATTACACTCATCCAGAAGG |
|  | Rev | TAATACGACTCACTATAGGGGTCACGATTTGTAAGAACCAGC |
| *Etv5* | Fwd | actgcatcgactcagaagtgcc |
|  | Rev | TAATACGACTCACTATAGGGggcaaagccctccgcgtaggg |
| *Alcam* | Fwd | cagattggagaggccctgcc |
|  | Rev | TAATACGACTCACTATAGGGGTCTCAGATTTATGATTGTTTTC |
| *Thra* | Fwd | TGGTGACCGAGGCGCACCGC |
|  | Rev | TAATACGACTCACTATAGGGCCTTCATGTGCAGGAAGCGGC |
| *Lypd1* | Fwd | CGGCTCTTCCTCCTCGCCGCTAC |
|  | Rev | TAATACGACTCACTATAGGGCAAGGAGCAGAGCGGTGGTTATC |
| *Cib2* | Fwd | GCACCAAACGTTGTGCCCATG |
|  | Rev | TAATACGACTCACTATAGGGAATAAGCTGAAACTTCCAGGATG |
| *Fpgs* | Fwd | AGCAGCATCCAAGCCTGCGTCC |
|  | Rev | TAATACGACTCACTATAGGGACCAGGTGCAGGCTGCCGGTG |
| *Tent5c* | Fwd | GAATGTGGAGCTGAAGTTTG |
|  | Rev | TAATACGACTCACTATAGGGGACTGGCTATATGGCACCGG |
| *Qsox1* | Fwd | TCAAGGAGCACTTCTCCCTG |
|  | Rev | TAATACGACTCACTATAGGGGGAAGCCGGGACGGCCCTTC |
| *Wsb1* | Fwd | CTGATGGACCATACAGAAGTTG |
|  | Rev | TAATACGACTCACTATAGGGCCACCACTTTTGATGGAGTAGG |
| *Rab18* | Fwd | GACGAGGACGTGTTGACCACG |
|  | Rev | TAATACGACTCACTATAGGGCATAGAACAATATCCACCACATG |
| *Atp6v1d* | Fwd | AGGCTCGCTTGAAGGGAGCTC |
|  | Rev | TAATACGACTCACTATAGGGCTCAAAGAGAAGGTCTTCATCC |
| *Skp1* | Fwd | CCTTCAATTAAGCTGCAGAGTTC |
|  | Rev | TAATACGACTCACTATAGGGCCACTGGTTCTCCTTACGTAC |
| *Psma6* | Fwd | CACATCACCATCTTTTCGCCC |
|  | Rev | TAATACGACTCACTATAGGGTCTCTGCCAGAGCTACAAGG |
| *Spcs1* | Fwd | CTGGACGTGTTCCGCTCC |
|  | Rev | TAATACGACTCACTATAGGGGCTTTTAGAATGCCTCTTTGG |
| *Mad2l1* | Fwd | GCCGCGCAGCTCAGCCGCGAG |
|  | Rev | TAATACGACTCACTATAGGGGTGAATCGTGGTAGTGAAGGAAC |
| *Slc2a8* | Fwd | GAAGCTAATGTTGAGGAGGAGGG |
|  | Rev | TAATACGACTCACTATAGGGACGCCTCAATCTGTTCCAGGG |
| *Klhl36* | Fwd | AGCTGGAGGCAGCTTTTCAAG |
|  | Rev | TAATACGACTCACTATAGGGACACACCAGCAATCGCTTTGGG |
| *Calm2* | Fwd | GCTGATCAACTGACAGAAGAGC |
|  | Rev | TAATACGACTCACTATAGGGCACCATCAATGTCTGCTTCCC |
| *Gpc3* | Fwd | TGACGTACGGGTGCCTGGCA |
|  | Rev | TAATACGACTCACTATAGGGCCCATCCTTGTTGTGCTGGT |
| *Gap43* | Fwd | AGCTCATAAGGCAGCCACCA |
|  | Rev | TAATACGACTCACTATAGGGGGCATTTTCTTGGTCCGCCT |
| *Rgs7bp* | Fwd | ACGACTGCAAGACGCTCGTC |
|  | Rev | TAATACGACTCACTATAGGGCTAATGGGTAGGGGGTCAAG |
| *Sstr5* | Fwd | ACTCTTCATGCTGGGCCTGC |
|  | Rev | TAATACGACTCACTATAGGGCACCTTGCTAGTCTGCATGTG |
| *Scn2a* | Fwd | CCGACTTGCCAGAATAGGCC |
|  | Rev | TAATACGACTCACTATAGGGTTCTGAGGGCATCCATCTCC |
| *Hs6st3* | Fwd | CAACCGCACGCTCAGGAACT |
|  | Rev | TAATACGACTCACTATAGGGCCACCGTGCAACCTGGCTAT |
| *Mdga1* | Fwd | ATGATGGGAAGCTGCGCCTG |
|  | Rev | TAATACGACTCACTATAGGGGCCGCGTCCAGTCAAAGTTG |
| *Chrnb3* | Fwd | GGGTCATGGACATATGATGGC |
|  | Rev | TAATACGACTCACTATAGGGCAAAGTGCTGTTCAGCCACATC |
| *Rtn1* | Fwd | AGCCTGTCAGAGGACGAGCT |
|  | Rev | TAATACGACTCACTATAGGGCAGGAGGCTGCCAAACACGA |
| *Pcp4* | Fwd | AGCGAGAGACAAGGAACCGG |
|  | Rev | TAATACGACTCACTATAGGGGGACTGGGACCCCGCTTTTT |
| *ChrnA5* | Fwd | GGTCTTGCAATCTCTCAGCTAG |
|  | Rev | TAATACGACTCACTATAGGGCTCATGCACAGCAGCTTGGG |
